# Stereotactic radiosurgery for residual rosette-forming glioneuronal tumor: a case report and literature review

**DOI:** 10.1007/s00701-026-06773-y

**Published:** 2026-02-02

**Authors:** Yung-Lin Hsiao, Huai-Che Yang, Chun-Fu Lin, Cheng-Chia Lee, Kang-Du Liou, Tzu-Chiang Peng

**Affiliations:** 1 School of Medicine, National Yang Ming Chiao Tung University, Taipei, Taiwan; 2 Department of Neurosurgery, Neurological Institute, Taipei Veterans General Hospital, 17 F, No. 201, Shih-Pai Road, Sec. 2, Beitou, Taipei, 11217 Taiwan ROC

**Keywords:** Rosette-forming glioneuronal tumor, Stereotactic radiosurgery, Gamma knife, Tumor, Fourth ventricle, Residual

## Abstract

Rosette-forming glioneuronal tumors (RGNT) are rare and novel World Health Organization grade I neoplasms that typically arise in the fourth ventricle and progress slowly. Surgical resection is the standard treatment. However, owing to their adherence to critical structures, complete resection is often not possible. The role of stereotactic radiosurgery (SRS) in the management of RGNT remains inconclusive. We present a case of tissue-confirmed RGNT successfully treated with SRS. A 24-year-old woman presented with diplopia and dysequilibrium and was subsequently diagnosed with a fourth ventricular tumor. Subtotal resection was performed at another hospital, and a tissue-based diagnosis of RGNT was made. After a multidisciplinary discussion and following the patient’s willingness, single-session SRS was prescribed at a marginal dose of 12 Gy. During the subsequent 66-month follow-up period, radiologic regression of the tumor with corresponding resolution of symptoms was noted. She remained neurologically intact at her last official visit. The treatment paradigm for residual RGNT remains elusive due to its scarcity and varied presentation. We have presented our preliminary experience with a residual RGNT that was managed with SRS, attaining long-term freedom from tumor progression. SRS may be a safe, effective, and durable treatment modality for patients with RGNT.

## Introduction

Rosette-forming glioneuronal tumor (RGNT) is a rare and novel intracranial World Health Organization grade I neoplasm that was first described by Komori et al. [3, 9, 11] They typically affect young adults and have a propensity to occur in the midline area, such as the fourth ventricle, cerebellar vermis, or pineal gland [19]. Owing to their scarcity, the clinical course remains enigmatic. The current understanding is based on retrospective series, and it is widely believed that they typically follow a more benign and indolent course. The long-term outcomes are generally favorable [2, 19]. However, an increasing number of serious studies have reported that it may undergo malignant transformation, disseminating through cerebrospinal fluid or invading nearby critical structures [1, 10]. Surgical evacuation is the standard treatment to relieve mass effect and establish tissue-based diagnosis. However, gross tumor resection may not be achieved because of their adherence to vital structures, such as the brain stem, dentate nucleus, and posterior inferior cerebellar artery [5]. The benefits of surgery should be weighed against the hazards of surgery, as aggressive advertent resection could potentially result in severe neurological morbidity. The role of radiotherapy in the management of RGNT remains unclear.

RGNT is characterized by a mixed solid and cystic component on magnetic resonance imaging. It typically appears iso- or hypo-intensity on T1 weighted imaging and hyper-intensity on T2 weighted sequence of magnetic resonance imaging (MRI). After the administration of contrast material, they exhibit heterogeneous contrast enhancement [2]. They generally have a clear demarcation with the peripheral, making them suitable targets for stereotactic radiosurgery (SRS). SRS has the advantages of a single session, precise targeting, and sharp fall-off irradiation. In addition, because most affected patients were young, radiosurgery could potentially avert long-term cognitive impairment and pituitary dysfunction attributed to traditional radiotherapy. However, relevant data regarding the effect and durability of SRS on RGNT are scarce [4]. We present a case successfully managed with SRS.

## Case presentation

**Fig. 1:**
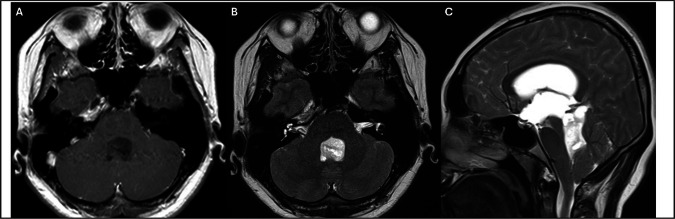
Contrast-enhanced axial T1-weighted (**A**) and T2-weighted (**B**) magnetic resonance images demonstrating a rosette-forming glioneuronal tumor prior to surgery. The lesion appears hypointense on T1-weighted images and hyperintense on T2-weighted sequences. It is primarily located in the fourth ventricle with periaqueductal extension (**C**). Mixed solid and cystic components are also identified

**Fig. 2:**
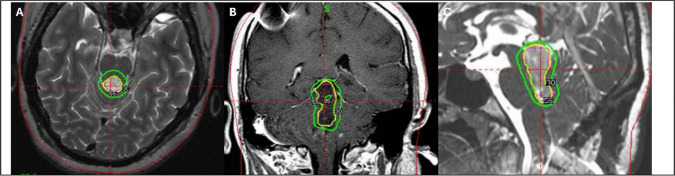
Gamma Knife radiosurgery planning for a fourth ventricle rosette-forming glioneuronal tumor. Axial (**A**), coronal (**B**), and sagittal (**C**) views of the dose-planning program demonstrate a 55% isodose line covering the target volume (3.86 cm^3^) with a peripheral dose of 12 Gy

**Fig. 3:**
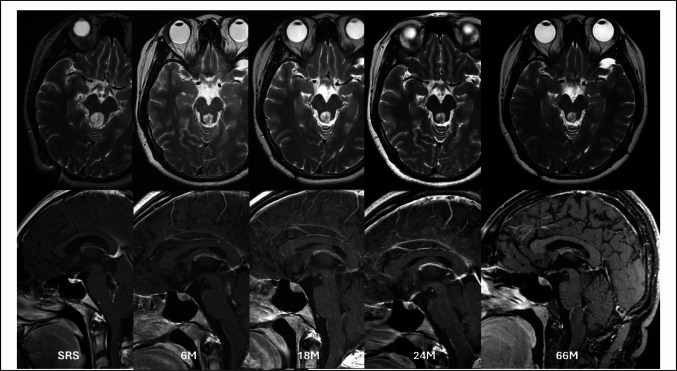
A 24-year-old female patient with a rosette-forming glioneuronal tumor of the fourth ventricle with periaqueductal extension. The patient initially underwent craniotomy for tumor evacuation. Residual tumor was evident on postoperative MRI, measuring 3.86 cm^3^, and she subsequently underwent stereotactic radiosurgery. Over the subsequent two years, the tumor volume continued to regress. The lesion remained stable at the most recent clinical follow-up, 66 months after stereotactic radiosurgery. No radiation-related adverse effects were observed throughout the follow-up period

A 24-year-old woman presented with progressive diplopia and gait instability, prompting a comprehensive neurological evaluation. Brain MRI revealed a well-circumscribed mass centered in the fourth ventricle with peri-aqueduct extension, causing obstructive hydrocephalus. The imaging features are shown in Fig. 1. To relieve hydrocephalus and secure a diagnosis, she underwent suboccipital craniotomy for tumor resection and ventriculoperitoneal shunt placement at another hospital. Subtotal removal was performed, and histopathological examination revealed RGNT. The patient was subsequently referred to our hospital for management. The case was presented at a multidisciplinary meeting. Given the lesion’s deep location and proximity to the brainstem, craniotomy for tumor evacuation was considered to be risky. In addition, the patient was unwilling to undergo another surgery. A single-session SRS targeting the residual tumor was performed in a shared decision-making manner. A marginal dose of 12 Gy prescribed to the 55% isodose line was delivered to the lesion. Collimator sizes of 4 mm and 8 mm were used. A total of 24 isocenters were applied, and the conformity index was calculated 82%. The procedure was performed using Gamma Knife Perfexion system. Radiosurgery planning is illustrated in Fig. 2. In subsequent imaging studies, the tumor regressed in size with corresponding relief of symptoms. Two years after SRS, the tumor volume significantly decreased. In the next 42-months period of follow-up period, the tumor was stationary without recurrence or progression. At the last official visit, the patient was neurologically intact and functionally independent. No adverse radiation effects were observed during the course of treatment. Serial imaging studies are shown in Fig. 3.

## Discussion

RGNTs are benign, slow-growing neoplasms that most often arise along the fourth ventricle and aqueduct [5]. Young women are more often affected, and the mean size was reported 2–3 cm at diagnosis [2, 19]. The symptomatology varies by location with associated mass effect, including headache, ataxia, visual disturbance, dysesthesia, and nausea. Even small lesions can produce disproportionate deficits when adjacent to the eloquent structures. Surgical resection is considered the standard treatment for RGNT. However, outcomes are not uniformly favorable due to its surgically challenging location and microscopic features [19]. In one cohort comprising 41 subjects, complete resection of RGNT was achieved in < 50% of cases, and postoperative neurological morbidity could occur in up to 47% of patients. Cranial nerve palsy (CN VI and VII) was the most commonly affected, which substantially hampered the long-term outcomes [19]. Despite pathologically indolence, this kind of tumor generally had more unfavorable outcomes after resection compared to other benign intracranial tumors [16, 17]. Given that RGNT is an exceedingly rare entity, there was controversy regarding the optimal therapeutic paradigm of residual tumors. Some authors believe that for residual tumors, an expectant approach with serial imaging studies is sufficient because of their slow-growing features. Repeated surgeries are performed until the patient became symptomatic. In contradistinction, more recent literature has found that the natural course of RGNT might not always be so protracted and sometimes they behave aggressively and deteriorated rapidly [10, 17]. Malignant transformation to glioblastoma has been described in numerous published reports, which highlighted the importance of lifelong imaging surveillance [6, 7, 18].

RGNT is characterized by hyper-intensity on T2 weighted imaging with well-defined margins that makes it a good candidate for SRS targeting. However, owing to its rarity, little is known about the effectiveness and durability of radiosurgery for RGNT. Ramos et al. was the first reporting on a case of recurrent fourth ventricle RGNT that was managed with radiosurgery [12]. The patient received single fraction SRS and demonstrated subsequent tumor regression during the follow-up period of seven years. In another case by Franzini, the author delivered a lower marginal dose of 13 Gy for patients with recurrent RGNT [4]. Although the tumor initially decreased in size, it flared up four years post-treatment. This subject received a second session of SRS with a marginal dose of 13.5 Gy, which had a more durable control for an additional seven years without tumor enlargement. Table 1 provides a review of the literature pertaining to the outcomes of SRS in the management of RGNTs. Although there is currently a lack of robust and convincing evidence, these representative cases imply that radiosurgery may be a potentially effective treatment modality for RGNT with a low risk of complications.

**Table 1:** Literature review of studies on the outcomes of SRS-treated RGNTs

Authors & Year	case	Location	Prior tx	Kind of radiosurgery	Radiation Tx Vol (cm^3^)	Margin Dose (Gy)	Max Dose (Gy)	Outcomes
Present study	1	Fourth ventricle and aqueduct	Suboccipital craniotomy	Gamma knife, single fraction	3.86	12	22.1	Tumor shrank in size and stabilized for more than 3.5 years
Shen et al., 2025 [15]	2	Aqueduct	Endoscopic third ventriculostomy and biopsy	Gamma knife, five fractions	16.57	5 × 5	55.6 in total	Tumor significantly reduced in volume three months after SRS
Franzini et al., 2023 [4]	3	Fourth ventricle	Suboccipital craniectomy	Two single-fraction Gamma knife, four years apart	NR	13 & 13.5	26 & 27	The tumor completely disappeared after the first session SRS. However, it recurred four years afterward. Second session SRS was delivered and the tumor decreased in size. Seven years after the last treatment, the tumor no longer existed in imaging
Ramos et al., 2018 [12]	4	Fourth ventricle	Suboccipital craniectomy	Gamma knife, single fraction	5.32	12	24	Tumor shrank in size and stabilized for seven years

*NR* Not report, *SRS* Stereotactic radiosurgery

Owing to its scarcity, SRS dosimetry is still under investigation. The current implicit concept of the therapeutic dose of RGNT in radiosurgery was primarily derived from other intracranial benign tumors. For benign meningiomas or schwannomas, a marginal dose of 12–18 Gy is usually delivered with gratifying outcomes [8, 13, 14]. Our case involved a biopsy-proven RGNT with residual disease, undergoing single-session SRS at a marginal dose of 12 Gy. The tumor maintained radiographic stability for 66 months without recurrence, progression, or additional interventions. There was no identified radiation induced change or delayed cyst formation throughout the course. Although our treatment dose was lower than that in previous studies, the patient attained favorable long-term outcomes with durable tumor control. Our preliminary experience lends credence to the concept that SRS could be a viable and safe alternative for patients with residual RGNT. A marginal dose of 12 Gy may be sufficient to achieve satisfactory tumor control. Further large-scale prospective studies with optimal patient selection are necessary to verify our findings.

## Conclusion

RGNT is a rare and locally invasive tumor. However, relevant literature in terms of the optimal management is sparse. Our preliminary experience demonstrates that radiosurgery may be served as an alternative treatment modality with favorable outcomes.

## Data Availability

The datasets relevant to this article are not publicly available due to privacy restrictions.

## Code availability

The data are not publicly available due to containing information that could compromise the privacy of research participants.
